# Long‐term climate warming and extreme cold events driving ecological shifts in a deep oligo‐mesotrophic lake

**DOI:** 10.1002/ece3.70052

**Published:** 2024-07-24

**Authors:** Kexin Zhu, Rong Wang, Hengshuai Qiu, Yu Zhao, Peng Xing, Jianan Zheng, Yanjie Zhao, Wenxiu Zheng, Xiangdong Yang

**Affiliations:** ^1^ State Key Laboratory of Lake Science and Environment, Nanjing Institute of Geography and Limnology Chinese Academy of Sciences Nanjing China; ^2^ University of Chinese Academy of Sciences Beijing China; ^3^ The Fuxianhu Station of Plateau Deep Lake Field Scientific Observation and Research Yuxi China; ^4^ Anhui Agricultural University Hefei China; ^5^ College of Geography and Ocean Sciences Nanjing University Nanjing China; ^6^ College of Urban and Environmental Sciences Hubei Normal University Huangshi China

**Keywords:** climate change, deep lake, eutrophication, internal nutrient cycle, regime shift

## Abstract

Deep lakes are critical for freshwater storage, yet they are struggling against major ecological issues from climate change and nutrient pollution. A comprehensive understanding of internal feedback mechanisms is crucial for regulating nutrients in these lakes. A five‐year study was conducted on the diatom community and environment in Lake Fuxian, China's largest deep freshwater lake, which is becoming eutrophic. The results indicate a shift in the diatom community from a stable state dominated by a single species to a rapid seasonal fluctuation, and there is a significant increase in diatom biomass. Specifically, stable stratification and low nutrient concentrations are limiting the growth of diatom biomass and maintaining the dominance of *Cyclotella*. Nutrients in the hypolimnion were replenished in the epilimnion during the extreme cold of winter, triggering a shift in the diatom community. This shift may imply that future climate change will exacerbate the positive feedback of hypoxia‐nutrient release of algal blooms, potentially triggering a regime shift in the ecosystem of the entire lake. This study underscores the fact that climate change alters the internal feedback mechanisms of deep lakes, reducing ecosystem stability, and provides a scientific basis for further clarification of protection measures for deep lakes.

## Graphical abstract

Our study analyzed a five‐year ecological transition in a deep lake by seasonal sampling, which used diatoms as indicators. The study found that long‐term warming and occasional extreme events profoundly altered the internal recycling mechanisms of the deep lake, which ultimately led to plankton community proliferation and regime shifts. The results suggest that future climate change may trigger dramatic regime shifts across more and more deep lakes at a global level, whose ecological security is under serious threat.

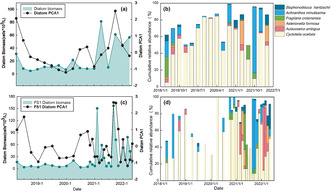

## 1 INTRODUCTION

Climate warming has resulted in higher water temperatures, earlier stratification, and increased thermal stability in deep lakes. Consequently, spring phytoplankton blooms are occurring earlier, and there is a proliferation of cyanobacteria (Paerl & Otten, 2013; Shimoda et al., 2011). The ultimate consequence of global warming could be a change in the mixing regime, which would profoundly alter the internal nutrient cycle of deep lakes (Mesman et al., 2021). Climatic warming reduces the vertical mixing range, leading to oxygen depletion in deeper water, the sequestration of phosphorus (P) in stagnant zones, and surface oligotrophication. The partial mixing (meromixis) is not permanent and can be partially reversed during exceptionally cold or windy winters. Holomixis (turnover) facilitates deep water reoxygenation and sudden phosphorus surges in surface water (Lau et al., 2020; Lepori et al., 2018), and P‐pulse events may trigger unexpected algal blooms (Yankova et al., 2017).

Freshwater lakes often experience widespread algal blooms. When temperatures or nutrient concentrations exceed critical thresholds, the water can quickly shift from clear to turbid with algal blooms (Scheffer et al., 2001). Ho et al. (2019) found that the peak summertime bloom intensity increased in 68% of the world's 71 largest lakes from 1987 to 2012. Although regime shifts have been extensively studied in lakes (Scheffer et al., 1993), these studies have mainly concentrated on shallow lakes, and it is still unclear how regime shifts occur in deep lakes. Some researchers have considered that eutrophication in deep lakes may be less severe than in shallow lakes (Qin et al., 2020). However, climate change and nutrient accumulation have caused regime shifts in deep lakes (Jilbert et al., 2020; Mesman et al., 2021). For example, Diamond Valley Lake (Los Angeles, maximum depth = 89 m, monomictic lake) experienced a regime shift due to an acute stressor (Gebremariam et al., 2021), shifting from a well‐oxygenated condition with low phytoplankton growth to a hypoxic, phytoplankton‐dominated turbid system (Gebremariam et al., 2021).

Diatoms are usually abundant and diverse in lakes, with individual species having specific habitat requirements. This characteristic is often used as an indicator of changes in hydrologic conditions and trophic levels (Hall & Smol, 1999; Rühland et al., 2015). Thackeray et al. (2008) examined how nutrient enrichment and climate change affect two spring diatom species. They found that the peak spring biomass of *Cyclotella* was related to earlier thermal stratification, while the peak biomass of *Asterionella* was linked to nutrient enrichment and lake warming. Diatom communities can serve as a characteristic indicator of how deep lakes respond to climatic and nutrient changes.

**FIGURE 1:**
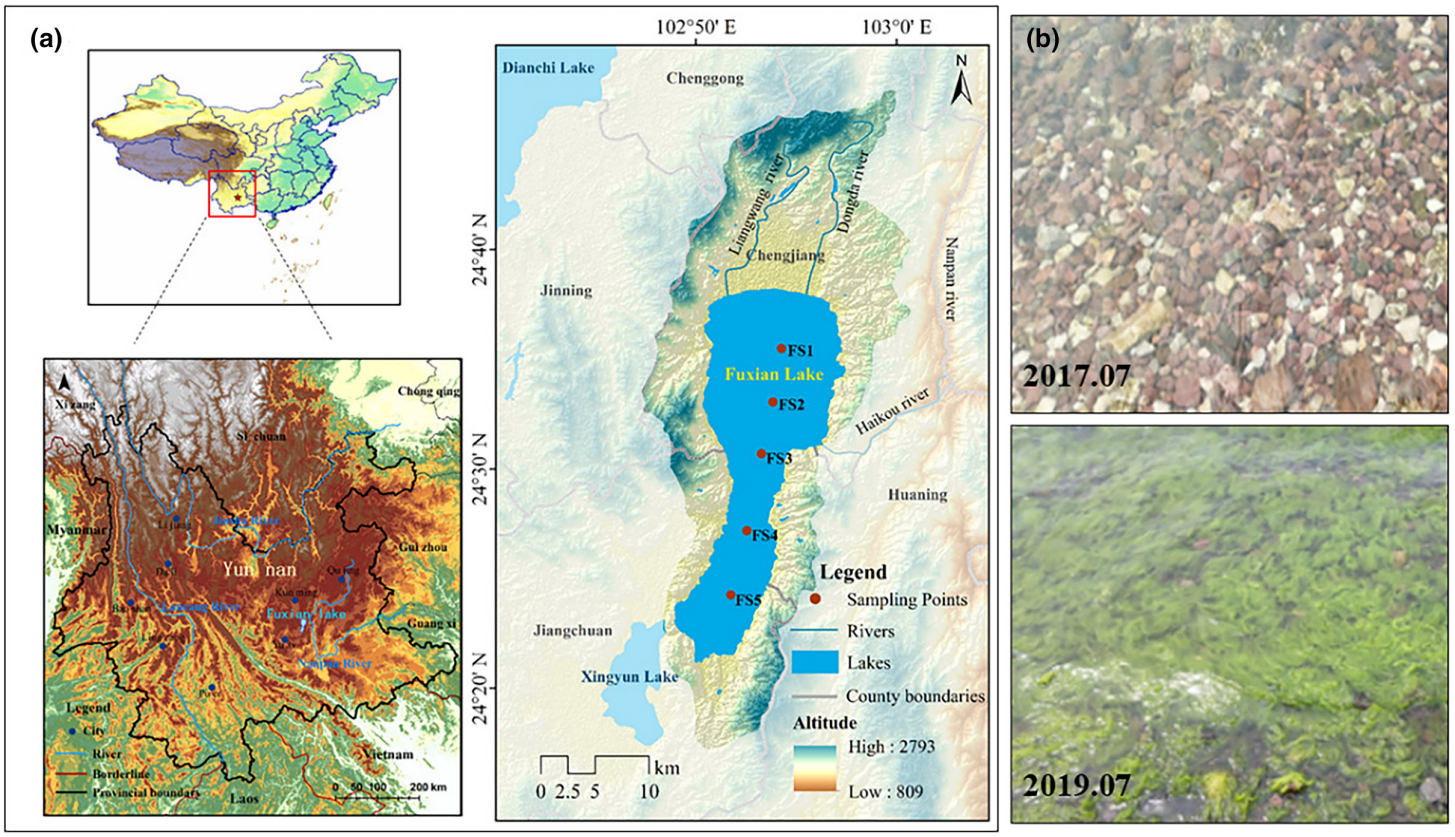
Lake Fuxian location (a) geography and sampling sites in Lake Fuxian; (b) water quality changes. Filamentous algae, such as *Cladophora* and *Spirogyra*, had significant growth in 2019 compared to 2017 (Photo provided by The Fuxianhu Station of Plateau Deep Lake Field Scientific Observation and Research).

Lake Fuxian is the largest deep freshwater lake in China. It has a water volume of 20.62 km^3^, representing approximately 9.16% of the total volume of China's freshwater lakes (Dai et al., 2017). The climate around the lake has warmed since the 1990s (Figure S1a), and human activities have exacerbated eutrophication in recent decades (Chen et al., 2019). The algae proliferation along the shores of Fuxian Lake has increased (Figure 1b). Strict watershed management has been implemented to maintain the water quality of Fuxian Lake, but the restoration efforts have not met expectations. Lake Fuxian is therefore a suitable example to study the way in which climate change and human activities affect deep lake ecosystem shifts and pollution management. We hypothesize that climate change alters the availability of internal nutrients to stimulate phytoplankton growth, counteracting the effects of watershed nutrient controls and potentially resulting in an ecosystem regime shift. To test this hypothesis, consecutive seasonal surveys were conducted in Lake Fuxian over a five‐year period, and the relationship between the abiotic environment and the diatom community was investigated. The main purpose of this study is to fill in a research gap on deep lake regime shifts in China and improve our understanding of regime shifts in deep lakes.

## 2 MATERIALS AND METHODS

### 2.1 Study site

Lake Fuxian (24.50° N, 102.89° E, 1721 m a.s.l) is located in Yuxi, Yunnan Province, approximately 53 km from Kunming (Figure 1a). It is a part of the Nanpan River, which is the mainstream of the Zhujiang River (Hu & Tao, 2023). This fault lake has a north–south orientation and a watershed area of 1084 km^2^. The lake is 212 km^2^ in size (Figure 1a **)** and has a maximum water depth of 155 m (Liu et al., 2014) and a water residence time of 167 years (An et al., 2022). The main rivers flowing into the lake include the Luju River, the Niumo River, and the Maliao River, while the Haikou River is the only outlet.

Lake Fuxian is a monomictic lake (a type of lake that experiences one mixing event annually.) with thermal stratification beginning in late March, peaking in August, and disappearing in December. The theoretical thickness of the thermocline is 22.4 m (Zhang et al., 2007). The water temperature of Lake Fuxian has increased since 1988 (Figure S1b), and the Secchi disk (SD) transparency has decreased by 1.52 m in 2011 compared to 1980 (Figure S1b). The total population in the area and the level of agriculture in the watershed have grown since the 1980s (Figure S1c), and nutrient loads entering the lake have increased significantly since 1998 (Figure S1d). A series of measures have been implemented in the region to improve water quality, including agricultural water conservation techniques, crop rotation, fallow land practices, and ecological protection (Yuxi Environmental Protection Agency and Yuxi Government official website). The watershed management has reduced nutrient inflow, slowing the increase of nutrient concentrations. However, the algae proliferation in Fuxian Lake has not been alleviated. In March 2018, a serious *Aphanizomenon flos‐aquae* bloom was noted in the northern part of Lake Fuxian, posing a significant threat to the safety of water quality in the entire lake (Li et al., 2020).

### 2.2 Physicochemical parameter analysis

We extracted temperature, precipitation, and wind speed data for the Kunming climate station between 2018 and 2022 from the National Climatic Data Center, a part of the NOAA (ftp://ftp.ncdc.noaa.gov/). Samples were collected quarterly from April 2018 to December 2020 (in January, April, July, and October). From January 2021 to May 2022, samples were collected monthly at FS1 and quarterly at FS2 to FS4. Total nitrogen (TN), nitrate (NO_3_^−^‐N), nitrite (NO_2_^−^‐N), ammonium (NH_4_^+^‐N), total phosphorus (TP), phosphate (PO_4_^3−^‐P), and chlorophyll a (Chl‐a) levels were analyzed according to standard methods (Ministry of Ecology and Environment of China, 2002). Dissolved oxygen (DO) and water temperature were measured in situ using a multiparameter water quality probe (YSI 6600, Yellow Springs, OH, USA). Dissolved oxygen data were collected four times per year (January, April, July, and October), and water temperature data were collected only in January and July.

### 2.3 Diatom analysis

Water samples (1 L) were collected from each sample site and treated with Lugol's iodine solution to fix phytoplankton species. The samples were then sedimented for 48 h and concentrated to 50 mL. We took 2 mL samples of condensed water prepared for diatom analysis heated with H_2_O_2_ (30%), followed by HCl (10%) to remove organic matter and carbonates, respectively. The field was traversed using an Olympus BX‐53 optical oil microscope to record the number of views and diatoms. Diatoms were identified using the Krammer and Lange‐Bertalot standard (1986, 1988, 1991a, 1991b), and diatom density in each sample was estimated by counting the number of diatoms per field of view to provide a measure of diatom biomass.

### 2.4 Statistical analysis

An average of the data collected from five sampling sites was used to represent the entire lake, with the FS1 data representing its deepest point. The data were transformed using the square root value to equalize variance before analysis. The length of the first axis of a detrended correspondence analysis (DCA) of the data was <3 SD (Braak et al., 1988), so a principal component analysis (PCA) was conducted to extract the major components of the diatom assemblages. PCA analyses were restricted to 26 diatom taxa with a minimum relative abundance of ≥2% that were present in ≥2 samples. This was done to minimize the impact of rare taxa. Redundancy analysis (RDA) was performed to quantify the relationship between the measured parameters and the six most dominant species at the FS1 site. Environmental variables were normalized after removing seasonal trends to remove the effects of seasonal dynamics and negative values before RDA analysis. PCA and RDA were performed using CANOCO 5.0 (Braak et al., 2012), and linear regression was used to fit the correlation between two factors, with a *p*‐value of <.05 signifying a good fit. The rate of change (RoC) (Pinek et al., 2020) indicates the rate of response of biotic or abiotic components to environmental changes. RoC curves were calculated using the R‐Ratepol package (Mottl et al., 2021).

## 3 RESULTS

### 3.1 Temporal dynamics of physiochemical parameters

**FIGURE 2:**
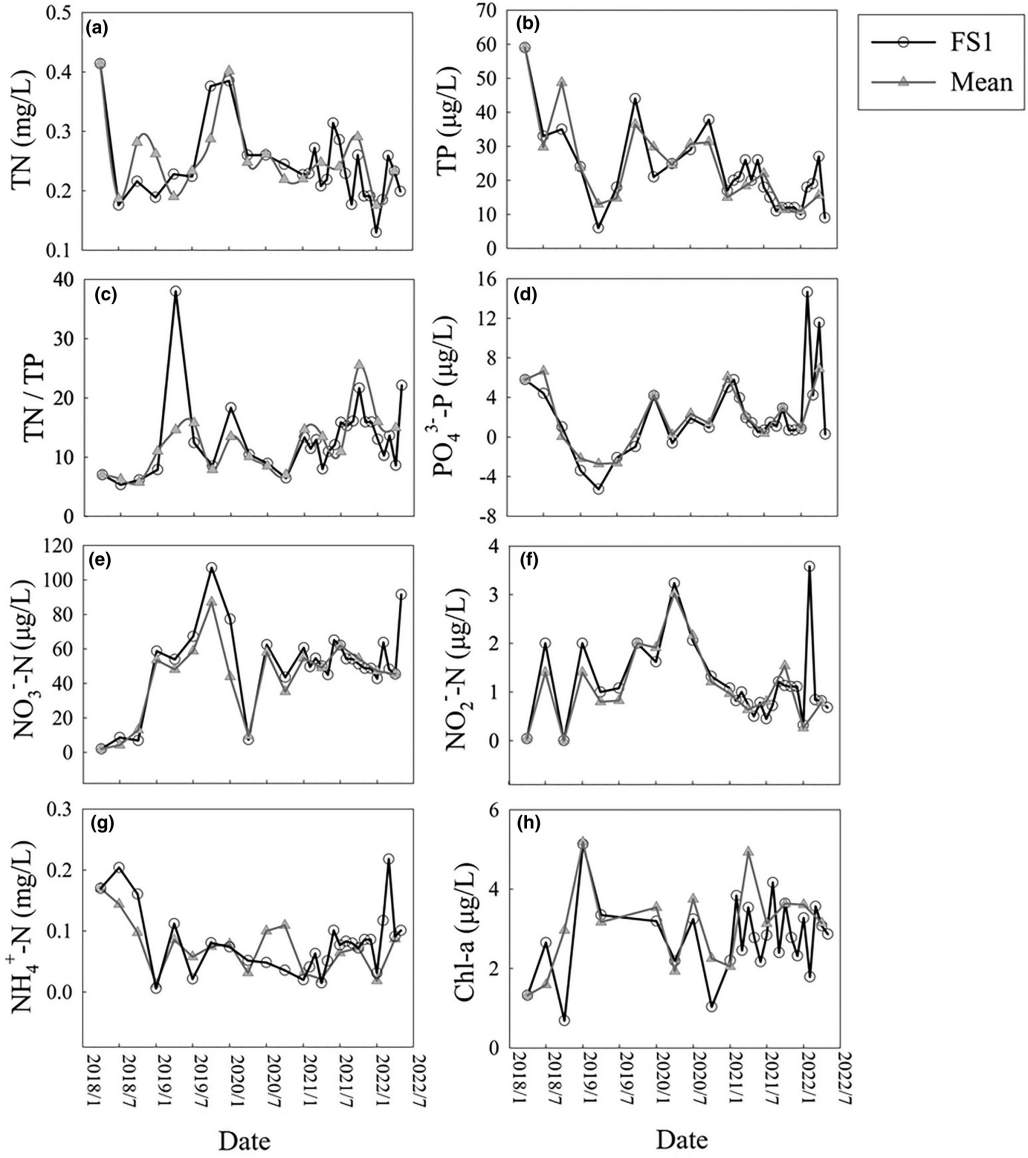
Inter‐annual dynamics of physiochemical parameters in Lake Fuxian The physiochemical parameters include (a) total nitrogen (TN); (b) total phosphorus (TP); (c) the mass ratio of TN/TP; (d) phosphate (PO_4_^3−^‐P); (e) nitrate (NO_3_^−^‐N); (f) nitrite (NO_2_^−^‐N); (g) ammonium (NH_4_^+^‐N); and (h) chlorophyll a (Chl‐a). The black open circles and gray triangles indicate the physiochemical characteristics of the FS1 site and the five‐point mean value, respectively. The timeline runs from April 2018 until May 2022. Negative phosphate values indicate that they were below the detection thresholds.

The physiochemical characteristics showed a roughly consistent temporal pattern across the entire lake (five sampling sites) and at the deepest site (FS1). There was no clear trend in TN (Figure 2a), fluctuating in the range of 0.33–0.18 mg/L. Only in April 2018 and January 2020 did TN concentrations exceed 0.40 mg/L. A significant decreasing trend in TP was observed, dropping from 59.0 μg/L in spring 2018 to 9.0 μg/L (FS1) in the spring of 2022 (Figure 2b). The TN/TP mass ratio increased, except for a few unusually high values (Figure 2c). The concentration of PO_4_^3−^‐P at the FS1 site showed a few high values in February and April 2022 (14.7 and 11.6 μg/L) (Figure 2d). Concentrations of NO_3_^−^‐N, NO_2_^—^N, and NH_4_^+^‐N remained stable over time, with an increase observed only in the spring of 2022 (Figure 2e–g). The Chl‐a values of FS1 fluctuated within a range of 1.8–4.2 (Figure 2h) since 2021.

### 3.2 Temporal dynamics of the diatom community

**FIGURE 3:**
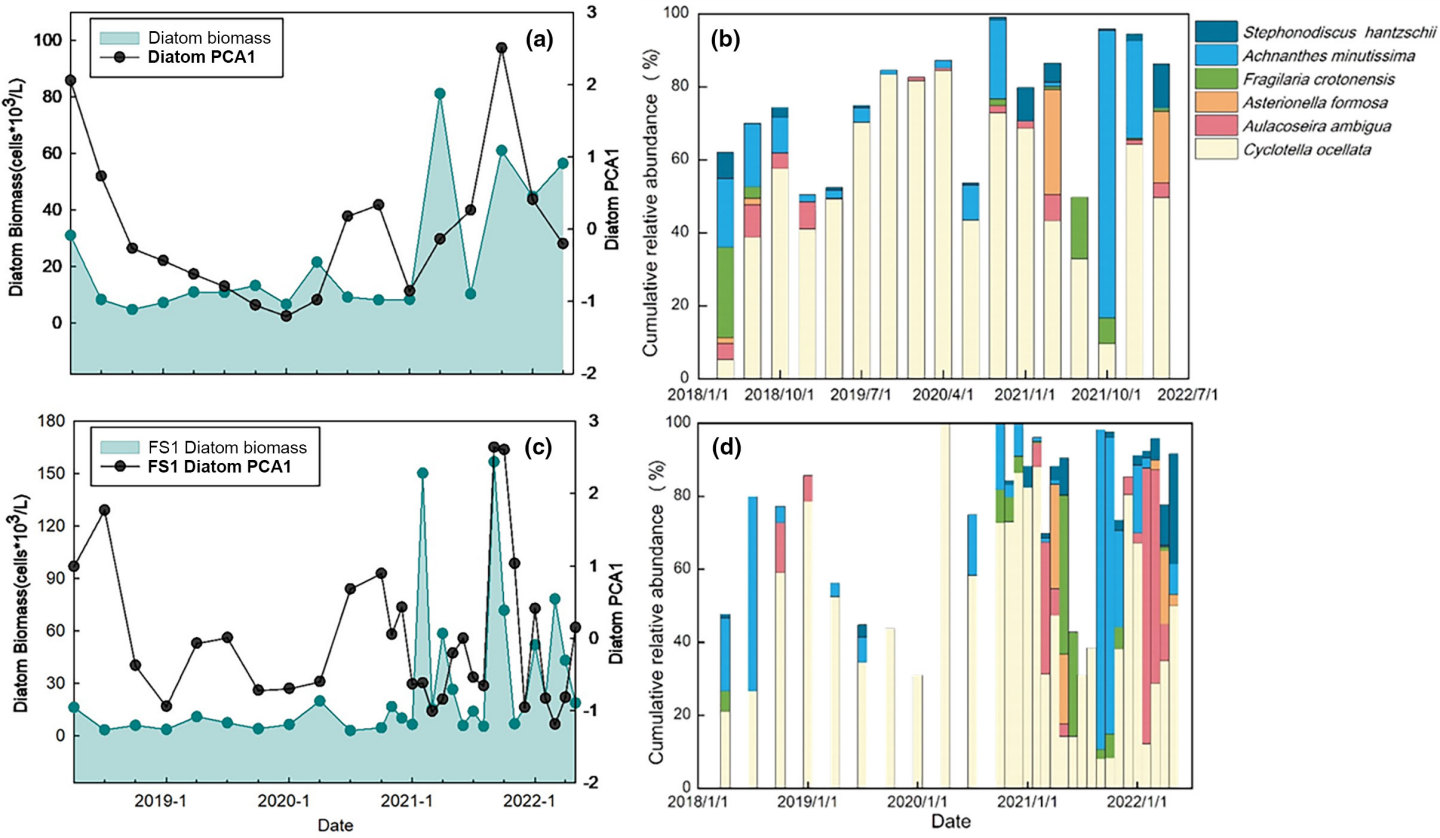
Shifts in diatom compositions (a) mean diatom biomass and PCA first‐axis score covering 5 sampling sites; (b) shifts in dominant diatoms covering 5 sampling sites; (c) diatom biomass and PCA first‐axis score at FS1; (d) shifts in dominant diatoms at FS1. In (a) and (c), black dots indicate PCA first‐axis score, and turquoise ranges and dots indicate diatom biomass.

The diatom community exhibited a consistent temporal pattern across the entire lake (five sampling sites) and at the deepest site (FS1) (Figure 3). The PCA first‐axis score (PCA1) for the diatom compositions was high in April and July 2018. The PCA1 remained low from October 2018 to July 2020, followed by a sudden increase and a more rapid change. Before 2021, diatom biomass was low, with concentrations below 35.00 cells × 10^3^ L^−1^, but it subsequently increased rapidly, reaching peaks in both spring and autumn. The mean biomass was 81.27 cells × 10^3^ L^−1^ in April 2021, and the diatom biomass at FS1 exceeded 150.00 cells × 10^3^ L^−1^ in February and September 2021.

A total of 61 diatom species within 20 genera were identified. Six genera, with *Fragilaria crotonensis*, *Asterionella formosa*, *Cyclotella ocellata*, *Stephonodiscus hantzschii*, *Aulacoseira ambigua*, and *Achnanthes minutissima*, represented the most dominant species, and a seasonal succession was observed. In April and July 2018, the diatom community was dominated by *F. crotonensis* and *A. minutissima*, but from October 2018 to February 2021, *C. ocellata* was the dominant species. The relative abundance of *A. minutissima* increased during the summer and autumn of 2020. After March 2021, the composition of diatoms began to change rapidly, and in Spring 2021, there was a notable increase in the relative abundance of *A. ambigua*, reaching 36.05%. In April 2021, the abundance of *A. formosa* increased, accounting for 28.53% of the diatom community, while *F. crotonensis* was most abundant from late Spring to early Summer (May–July) in 2021. A significant increase in *A. minutissima* was observed in the Autumn of 2021, with a relative abundance of 87.65% in September and 81.32% in October. In February 2022, *A. ambigua* increased rapidly and became dominant, with a maximum percentage of 75.47%. A significant rise in the relative abundance of *A. formosa* was observed in April 2022, reaching 20.07%, and this was followed by a gradual increase in *F. crotonensis*. In May 2022, the relative abundance of *S. hantzschii* was 30.21%.

### 3.3 Redundancy analysis results

**FIGURE 4:**
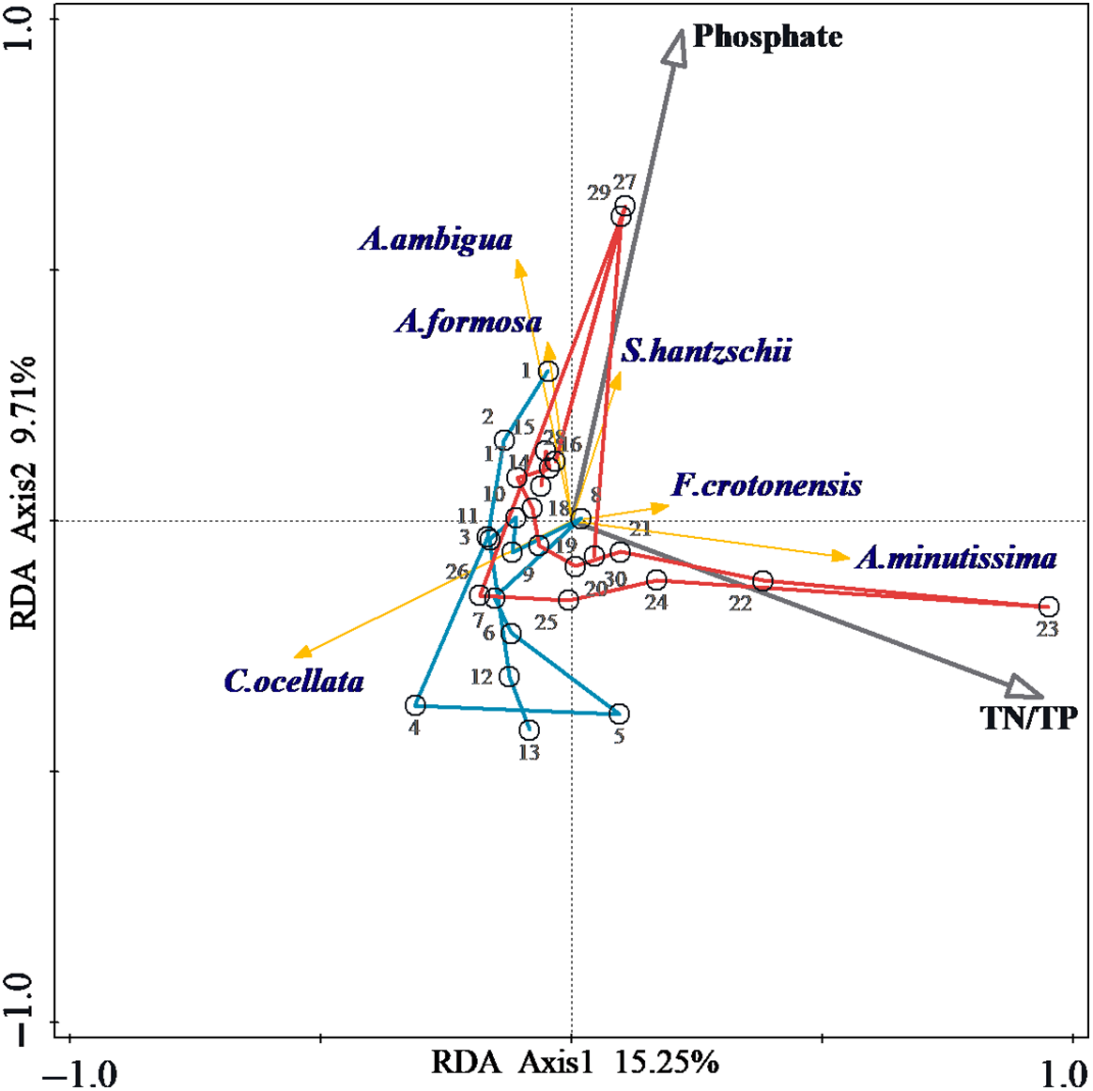
RDA ordination plot. The blue line shows data points for 2018–2020, and the red line shows data points for 2021–2022.

The results of RDA analysis showed that TN/TP changes played the most significant role in diatom succession, followed by lake phosphate concentrations (Figure 4). RDA axes 1 and 2 explained 25.0% of the cumulative variance in the species–environment relationship. TN/TP had a strong relationship to axis 1 and was significantly correlated with *A. minutissima*. Phosphate had a strong relationship to axis 2 and was significantly correlated with *A. ambigua*, *A. formosa*, and *S. hantzschii*. The RDA results showed that alterations to the diatom assemblage were driven by two factors and shifted more drastically after 2021.

## 4 DISCUSSION

### 4.1 Temperature driven nutrient cycling in Fuxian Lake

The TP concentrations in Lake Fuxian did not show any seasonal variations, although they showed a decreasing trend. Since 2016, control measures in the lake watershed have been strengthened, with a focus on addressing agricultural pollution and preventing eutrophication. These measures helped to minimize the influence of seasonal variations in climate on TP concentration, but phosphate levels increased suddenly during the winter of 2021–2022 (Figure 2d), which may indicate the effects of internal P release.

The annual mean air temperature of Lake Fuxian has increased significantly (Figure S1a), and the effect of this has been to stabilize thermal stratification. The temperature difference between the surface and bottom in 2019 and 2020 was about 2°C (Figure S5). Stratification impedes the exchange of materials between the hypolimnion and epilimnion (Wang et al., 2017), resulting in severe hypoxia in the hypolimnion (Figure S3). Anoxia at the bottom of the lake creates a suitable environment for nutrient release from sediments, while Duan et al. (2023) observed higher phosphorus concentrations in the deeper water of Lake Fuxian.

Extreme cold can destabilize deep water, leading to exceptionally strong mixing events (Holzner et al., 2009). In the winter of 2022, the coldest year since 2018 (Figure S2), extremely cold temperatures led to deep water mixing. Temperature differences between the surface and bottom decreased (Figure S5), and dissolved oxygen was replenished from the epilimnion to the hypolimnion (Figure S3) and nutrients to the epilimnion. These results closely match the findings that fluctuations in winter temperature affect DO and nutrient cycling in deep lakes (Finsinger et al., 2014).

### 4.2 Diatom community instability rising

RDA analyses have identified phosphate and TN/TP as the two main factors affecting changes in diatom communities. Before 2021, decreased nutrient concentrations and stable stratification allowed for the dominance of small‐celled *C. ocellata* (Yan et al., 2018). After 2021, higher TN/TP concentrations promoted the proliferation of *A. minutissima*, which prefers high temperatures and nitrogen (Paul et al., 2010; Winter & Duthie, 2000). Water turnover driven by extreme cold adds to the mixing hydrodynamics, which favors the proliferation of *A. ambigua* (Rühland et al., 2008). Meanwhile, epilimnion nutrients were replenished, promoting an increase in trophic species including *A. formosa*, *F. crotonensis*, and *S. hantzschii* (Kirilova et al., 2008; Saros et al., 2005).

Diatom biomass increased with shifts in community structure. These changes were associated with the water turnover, which can recycle nutrients, including those released from the sediment under anoxic conditions. Nutrient limitations on algal production were offset by increased concentrations of nutrients, a phenomenon commonly observed in deep lakes (Peeters et al., 2012). The turbulent state of rapid seasonal fluctuation has replaced the stable state of *C. ocellata* as the dominant species (Figure 3). The ROC values indicating the RoC in diatom biomass and community structure also showed a significant increase (Figure S4). All these changes indicate increased instability within the diatom community. In studies at longer time scales, it has been found that lakes underwent long periods of instability or fluctuation prior to regime shifts (Spanbauer et al., 2014). Deep lakes may have a much lower nutrient threshold compared to shallow lakes, and the range of nutrient concentrations at which two stable states can coexist may be narrower (Scheffer & van Nes, 2007).

### 4.3 Regime shift risk in the deep lake ecosystem

Climate model projections suggest that warming will continue in the short‐term future (Brown & Caldeira, 2017). The extreme cold in China with a return period of 10 years becomes an event that occurs every 67 years based on a warming of 1.5 °C (Sun et al., 2019). This means that in the future, the stratification period of Lake Fuxian may continue to lengthen, causing the anoxic zone at the bottom to expand and intensify the release of nutrients. During extremely cold winters, a large amount of nutrients that have accumulated in the hypolimnion will move to the epilimnion because of lake turnover. This may generate a nutrient pulse effect. Unfortunately, pulse nutrient disturbances amplify the detrimental effects of nutrients on phytoplankton proliferation (Zhao et al., 2022). We hypothesize that these processes amplify the positive feedback loop (bottom hypoxia‐nutrient release‐algal bloom‐exacerbated hypoxia) in Lake Fuxian, and regime shifts throughout the entire lake may occur if positive feedback encourages algal blooms until they exceed the thresholds for ecosystem stability (Scheffer & Carpenter, 2003).

While there is a great deal of uncertainty about the future of climate‐driven algal blooms in Lake Fuxian, other deep lakes have also experienced regime shifts. Studies on Diamond Valley Lake have shown that interactions between hypoxia, internal P loading, and seasonal cyanobacterial blooms act as mechanisms that perpetuate the new alternative state (Gebremariam et al., 2021). According to Istvánovics et al. (2022), it is unlikely that reducing the external load will keep pace with increases in internal loading. With continued warming and associated changes in internal processes, it is possible that we will see dramatic regime shifts across more and more deep lakes at a global level. The management of deep lakes requires the close monitoring of internal nutrient release, and the increasing challenge of climate change also needs to be considered.

## 5 CONCLUSIONS

Anthropogenic efforts to reduce nutrients have succeeded in stabilizing or even decreasing TN and TP concentrations. However, our study emphasizes the profound effects of temperature change on deep lakes. Prolonged warming leads to an increase in the release of nutrients in the lake, while occasional cold temperature events cause fluctuations in epilimnion nutrient concentrations, amplifying their effects on the proliferation of phytoplankton. Temperature‐driven shifts in nutrient availability have reduced the stability of the ecosystem and increased the risk of ecosystem regime shifts. The characteristics of Lake Fuxian's response to changes in nutrients and temperature can provide insights into other deep lakes, and reducing the impacts of nutrient releases in the context of climate change is a critical issue for the management of deep lakes. High‐frequency monitoring and aquatic community analysis may provide an early warning of regime shifts and help in the development of advanced deep lake management programs.

## AUTHOR CONTRIBUTIONS

**Kexin Zhu:** Conceptualization (lead); formal analysis (lead); investigation (lead); methodology (lead); visualization (lead); writing – original draft (lead); writing – review and editing (lead). **Rong Wang:** Conceptualization (supporting); formal analysis (supporting); funding acquisition (lead); investigation (supporting); writing – review and editing (supporting). **Hengshuai Qiu:** Formal analysis (supporting); funding acquisition (supporting); investigation (supporting); visualization (supporting). **Yu Zhao:** Formal analysis (supporting); methodology (supporting); writing – review and editing (supporting). **Peng Xing:** Funding acquisition (supporting); writing – review and editing (supporting). **Jianan Zheng:** Formal analysis (supporting); methodology (supporting). **Yanjie Zhao:** Writing – review and editing (supporting). **Wenxiu Zheng:** Writing – review and editing (supporting). **Xiangdong Yang:** Funding acquisition (supporting).

## FUNDING INFORMATION

This work was supported by the National Key Research and Development Program of China (2022YFF0801104) and the National Natural Science Foundation of China (NSFC, 42330511, 42171163, and U2102216). R.W. acknowledges the financial support of the Youth Innovation Promotion Association of the Chinese Academy of Sciences (award Y2021086) and the Youth Scientists Group at the Nanjing Institute of Geography and Limnology, CAS (2021NIGLAS‐CJH03).

## CONFLICT OF INTEREST STATEMENT

We declare there is no conflict of interest.

## Supporting information


Figure S1.‐S5.

Data S1.

## Data Availability

The data that support the findings of this study are openly in the Supplementary materials.
