# Supplementary material for: Long‐term climate warming and extreme cold events driving ecological shifts in a deep oligo‐mesotrophic lake
Source: Ecol Evol. 2024 Jul 24;14(7):e70052. doi: 10.1002/ece3.70052 (PMC11268934; doi:10.1002/ece3.70052)
Supplement: Supplementary file 1 — Figure S1.‐S5. [file ECE3-14-e70052-s001.docx]

**Figures** **S1**

**Figure S1: Long-term environmental changes in Lake Fuxian** (a) annual mean air temperature and annual precipitation, along with their anomalies (National Meteorological Information Center, http://data.cma.cn/); (b) changes in annual mean water temperature and Secchi disk (SD) transparenc(Gao et al., 2013); (c) total population and fertilizer consumption in the Lake Fuxian watershed covering the three counties (Yunnan Statistical Yearbook and Yuxi Statistical Bureau); (d) Annual inflow of TN and TP from major rivers into the lake(Gao et al., 2013).

**References**

Gao, W., Chen, Y., Xu, M., Guo, H., & Xie, Y., (2013). Trend and driving factors of water quality change in Lake Fuxian(1980-2011). *Journal of Lake Sciences*, 25: 635-642. <https://doi.org/10.18307/2013.0503>.

**Figures** **S2**

**Figure S2: Temporal trends in climate variables** (a) Temperature; (b) Precipitation; (c) wind speed.

**Figures** **S3**

**Figure S3: Dissolved oxygen in the water column**

**Figures** **S4**

**
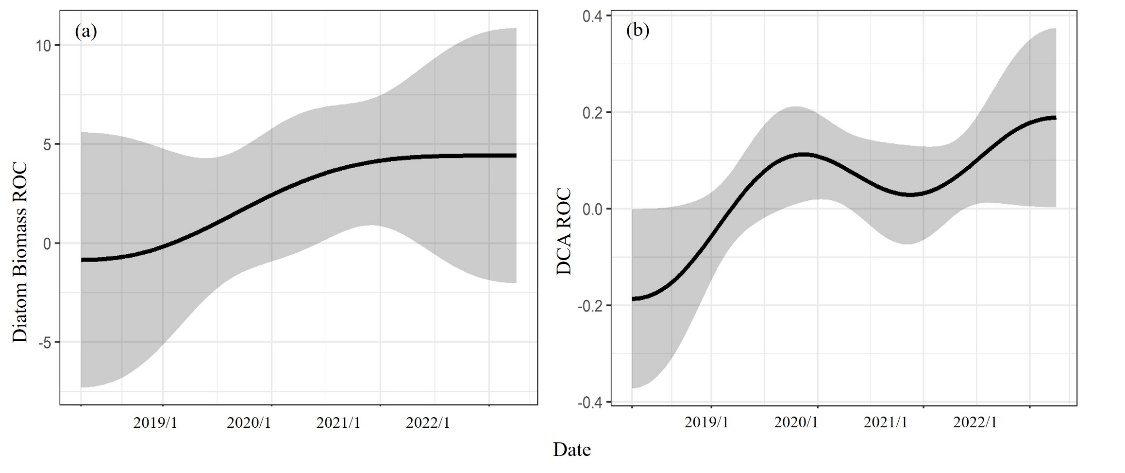
**

**Figure S4: Rate of change** the grey shaded area is the 95% confidence interval.

**Figures** **S5**


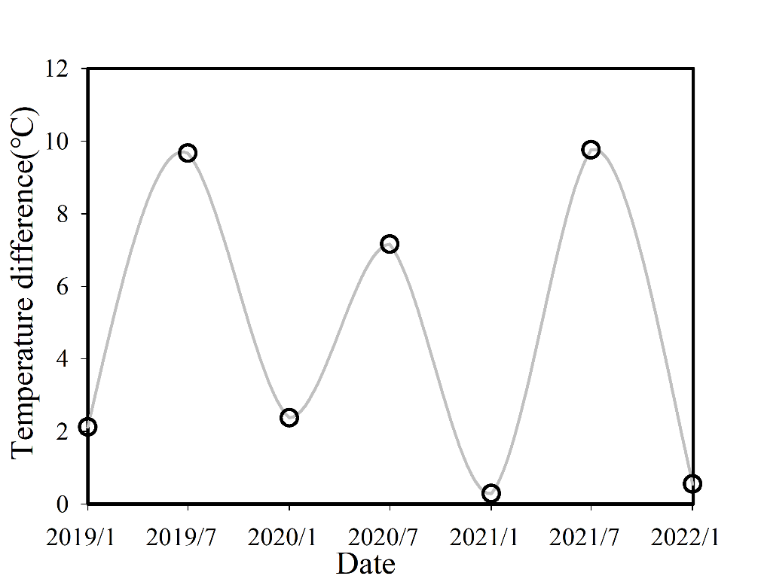


**Figure S5: Temperature difference between surface and bottom**
